# Development of a practical dietitian road map for the nutritional management of phenylketonuria (PKU) patients on pegvaliase

**DOI:** 10.1016/j.ymgmr.2021.100771

**Published:** 2021-05-25

**Authors:** Júlio César Rocha, Heather Bausell, Amaya Bélanger-Quintana, Laurie Bernstein, Hülya Gökmen-Özel, Alexandra Jung, Anita MacDonald, Fran Rohr, Esther van Dam, Margret Heddrich-Ellerbrok

**Affiliations:** a Nutrition & Metabolism, NOVA Medical School, Faculty of Medical Sciences, University of Lisbon, Lisbon, Portugal; b Reference Centre of Inherited Metabolic Diseases, Centro Hospitalar Universitário de Lisboa Central, Lisbon, Portugal; c Center for Health Technology and Services Research (CINTESIS), Porto, Portugal; d Division of Clinical Nutrition & Genetics, Ann & Robert H. Lurie Children's Hospital of Chicago, Chicago, IL, USA; e Metabolic Disease Unit, Hospital Ramon y Cajal, Madrid, Spain; f Department of Pediatrics Section of Clinical Genetics and Metabolism, Aurora, Children's Hospital Colorado, University of Colorado, Anschutz Medical Campus, Aurora, CO, USA; g Nutrition and Dietetics Department, Faculty of Health Sciences, Hacettepe University, Ankara, Turkey; h Competence Center for Rare Metabolic Diseases, Charité – University Hospital Berlin, Berlin, Germany; i Department of Dietetics, Birmingham Women's and Children's Hospital, Birmingham, UK; j Met Ed, Boulder, CO, USA; k Department of Dietetics, University of Groningen, University Medical Center Groningen, Beatrix Children's Hospital, Groningen, The Netherlands; l University Children's Hospital, University Medical Center Hamburg-Eppendorf, Hamburg, Germany

**Keywords:** Phenylketonuria, PKU, Phenylalanine, Pegvaliase, Nutrition, Dietitian, Nutritionist, BMI, body mass index, DRI, dietary reference intake, DXA, dual-energy x-ray absorptiometry, EMA, European Medicines Agency, FDA, Food and Drug Administration, LBP, low blood phenylalanine, PAH, phenylalanine hydroxylase, PAL, PEGylated recombinant phenylalanine ammonia lyase, Phe, phenylalanine, PKU, phenylketonuria, QoL, quality of life

## Abstract

**Background:**

The metabolic dietitian/nutritionist (hereafter ‘dietitian’) plays an essential role in the nutritional management of patients with phenylketonuria (PKU), including those on pegvaliase. Currently, more educational support and clinical experience is needed to ensure that dietitians are prepared to provide optimal nutritional management and counselling of pegvaliase-treated patients.

**Methods:**

Via a face-to-face data-review meeting, followed by a virtual consolidation meeting, a group of expert dietitians and one paediatrician discussed and developed a series of recommendations on the nutritional evaluation and management of patients receiving pegvaliase. The consensus group consisted of 10 PKU experts: six dietitians and one paediatrician from Europe and three dietitians from the US. One European and three US dietitians had experience with pegvaliase-treated patients.

**Results:**

The consensus group recommended that a physician, dietitian and nurse are part of the pegvaliase treatment team. Additionally, a psychologist/counsellor should be included if available. Practical proposals for the nutritional evaluation of pegvaliase-treated patients at baseline, during the induction and titration phases and for long-term maintenance were developed. The consensus group suggested assessment of blood Phe at least monthly or every 2 weeks in the event of low blood Phe (i.e., blood Phe <30 μmol/L). It may be appropriate to increase blood Phe monitoring when adjusting protein intake and/or pegvaliase dose. It was recommended that natural protein intake is increased by 10–20 g increments if blood Phe concentrations decrease to <240 μmol/L in patients who are not meeting the dietary reference intake for natural protein of 0.8 g/kg. It was proposed that with pegvaliase treatment blood Phe levels could be maintained <240 μmol/L but more evidence on the safety of achieving physiological blood Phe levels is necessary before any recommendation on the lower blood Phe target can be given. Finally, both patients and dietitians should have access to educational resources to optimally support patients receiving pegvaliase.

**Conclusion:**

This practical road map aims to provide initial recommendations for dietitians monitoring patients with PKU prescribed pegvaliase. Given that practical experience with pegvaliase is still limited, nutritional recommendations will require regular updating once more evidence is available and clinical experience evolves.

## 1 Introduction

Phenylketonuria (PKU) is a rare autosomal recessive disorder caused by deficiency in activity of the hepatic enzyme, phenylalanine hydroxylase (PAH) [1]. Without treatment, it results in elevated blood and brain phenylalanine (Phe) concentrations, leading to neurocognitive and neuropsychological complications [1,2]. American and European guidelines recommend lifelong treatment and follow-up although their upper blood Phe targets vary (in patients aged ≥12 years ≤360 and 600 μmol/L, respectively) [2,3]. Dietary restriction of Phe, maintaining blood Phe within treatment range, can prevent severe complications when initiated immediately after diagnosis, usually soon after birth as PKU is identified via newborn screening programmes [2,3]. However, dietary management poses a significant burden to patients with PKU, resulting in a progressive deterioration in dietary adherence and a subsequent increase in blood Phe concentrations [4,5]. Sapropterin dihydrochloride (sapropterin) was the first non-dietary pharmacological therapy for PKU [6,7]. Although effective in lowering blood Phe concentrations and increasing Phe tolerance, it is mainly used in conjunction with a Phe-restricted diet and in patients with residual PAH activity [[8], [9], [10]]. The treatment landscape of PKU has further expanded with the approval of pegvaliase, an enzyme substitute therapy consisting of a PEGylated recombinant phenylalanine ammonia lyase (PAL) isolated from the cyanobacterium *Anabaena variabilis* that converts Phe to ammonia and trans-cinnamic acid [11]. In May 2018, the Food and Drug Administration (FDA) approved the use of pegvaliase for the treatment of adult patients with PKU with blood Phe >600 μmol/L [12]. In May 2019, the European Medicines Agency (EMA) approved pegvaliase for the treatment of PKU patients aged ≥16 years with blood Phe >600 μmol/L [13]. Consensus recommendations for pegvaliase were developed in 2018 by US healthcare professionals who were involved in the clinical development programme [14]. These consensus recommendations defined that the treatment goal of pegvaliase is to provide lifelong reduction of blood Phe and to normalise diet while maintaining blood Phe below guideline recommended thresholds [14]. Given the central role of metabolic dietitians/nutritionists (hereafter ‘dietitian’) in the management of patients with PKU, this paper aims to develop a practical road map for dietitians who are managing patients with PKU treated with pegvaliase. These dietary recommendations are based on expert opinion and should have regular review when real-world evidence accumulates with evolving clinical pegvaliase experience. Therefore, these dietary recommendations are intended as a starting point for dietitians who are caring for patients with PKU on pegvaliase.

## 2 Methods

A group of experienced dietitians and one paediatrician (hereafter ‘consensus group’) working with PKU attended a face-to-face data-review meeting, followed by a virtual consolidation meeting, to discuss the nutritional management of PKU patients before and during treatment with pegvaliase.

At the time, the only European country which had experience with pegvaliase was Germany. Therefore, one German dietitian who was experienced in treating and managing patients on pegvaliase was included to bring the EU perspective on treatment with pegvaliase. The other six EU PKU experts, who did not have experience with pegvaliase, are internationally recognised for their experience in PKU and most of them are part of the European Nutritionist Group on PKU. Additionally, three US dietitians with proven long-term experience in PKU and in treating and managing diet for patients on pegvaliase joined the meeting. Both meetings aimed to use insights from these EU and US PKU experts to develop consensus on the nutritional evaluation before initiation of pegvaliase and to develop recommendations for dietary adjustments during treatment with pegvaliase.

### 2.1 Face-to-face dietitian meeting

In November 2019, eight dietitians (six EU and two US dietitians) attended the face-to-face meeting. During the meeting, the pegvaliase clinical trial data and prescription labels were reviewed and topics concerning the nutritional management of PKU patients in the context of pegvaliase were discussed. Discussion was structured and guided by presentations with questions formulated by three of the expert dietitians. Detailed minutes of the meeting were taken and 17 draft recommendations were formulated.

### 2.2 Virtual consolidation meeting

Recommendations were developed in five areas of pegvaliase treatment: the multidisciplinary team (2 statements), nutritional status evaluation and follow-up (4 statements), diet and protein intake (7 statements), blood Phe target range (2 statements) and education (2 statements).

In March 2020, a secure online-platform was set up using the virtual engagement tool, *Within3* (https://www.within3.com/), aiming to work towards consensus recommendations for the nutritional management of pegvaliase-treated patients. On the platform, the PKU experts could share their opinion on each statement and respond to each other's comments. Participants could log in as often as desired at any time during a 4-week commenting period. Except for two EU dietitians, all attendees of the face-to-face meeting participated in the virtual meeting. Additionally, one EU paediatrician and one US dietitian who were not present at the face-to-face meeting participated in the virtual platform discussion.

### 2.3 Practice recommendations

The practice recommendations and outcomes of the discussions from both meetings are described. Practice recommendations on the clinical approach for treating patients with pegvaliase were initiated by the dietitians with pegvaliase experience (three US and one EU dietitian) while the other experts considered each of these using their experience working with PKU within the EU to ensure good applicability in European centres. Although the statements are based on expert opinion and available literature only, they aim to give practical guidance to dietitians. These practice recommendations are minimum care requirements and consider different hospital resources and infrastructure within the same or different countries.

## 3 Results

### 3.1 A multidisciplinary team to support patients with PKU on pegvaliase

It is important to establish key members of the multidisciplinary team supporting patients on pegvaliase and to systematically collect data to measure the full impact of introducing pegvaliase as a treatment. The consensus group agreed that the core pegvaliase treatment team should consist of a physician, a dietitian and a nurse (either a registered nurse or a nurse practitioner). It also strongly recommended that a psychologist/counsellor should be an integral part of the core pegvaliase treatment team emphasising the importance of assessing the neurocognitive and psychological status of pegvaliase-treated patients, although it was recognised some clinics may not have access to these resources. Considering the challenges imposed by this new pharmacological treatment, a detailed analysis of the patient's expectations may justify the need for a psychologist/counsellor in the team. If resources are available, a social worker, home support worker and coordinator should be part of the multidisciplinary treatment team. Table 1 provides an overview of all specialists and their respective roles within the multidisciplinary team to ultimately ensure optimal and safe use of pegvaliase for the treatment of patients with PKU.

**Table 1:** Proposed composition of the multidisciplinary team at centres treating PKU patients with pegvaliase.

Specialty	Responsibilities
**Consensus group recommendations about the composition of the multidisciplinary teams at centres treating PKU patients with pegvaliase**	


Physician	- Diagnosis and decisions on appropriate therapeutic approach, considering potential adverse events
	- Pegvaliase prescription
	- Patient assessment prior to receiving pegvaliase
	- Titration of pegvaliase dose and assessment of patient progress during follow-up clinic visits
	- Management of adverse reactions

Dietitian/nutritionist	- Key role in monitoring and assuring adequate nutritional status of patients
	- Detailed food pattern analysis pre- and post-pegvaliase
	- Nutrition re-education as a strategy to reduce the risk of lifestyle-related comorbidities
	- Assessment of nutritional intake
	- Interpretation of blood Phe results in conjunction with dietary evaluation
	- Assessment of patient progress during follow-up clinic visits
	- Contribute to decisions on appropriate therapeutic approach, based on individual (dietetic) patient status/needs
	- Point of contact for the patient in terms of blood Phe reporting and diet education

Nurse	- Education and practical support of patients on pegvaliase injections
	- Point of contact for the patient in terms of managing drug titration and side effects
	- In some countries, nurse practitioners can also prescribe medication and assess patient's progress during follow-up clinic visits
	- Underline the global advice given by the physician and dietitian regarding treatment adherence, administration and dietary management


***If resources are available,*****the following specialties should be included in the multidisciplinary team:**	


Psychologist/counsellor (highly recommended)	- Surveillance and follow-up on any neurocognitive/psychological deficits
	- Perform neurocognitive testing and quality of life assessments
	- Patient counselling and support
	- Monitor patient's fears and help manage their expectations

Social worker	- Assessing individual patients' socioeconomical needs and support system, and providing assistance in finding solutions in these areas
	- Advising and supporting patients in contacting the necessary service providers/agencies

Home support worker	- Home support with blood sampling, treatment, social challenges and pregnancy management
	- Can be any profession depending on availability, although a nurse would be able to provide education and support regarding injections and potential adverse events

Coordinator	- Administrative assistant as central point of contact for e.g., organising appointments
	- Especially valuable at treatment initiation to coordinate appointments with the multidisciplinary team

Phe: phenylalanine, PKU: phenylketonuria.

### 3.2 Nutritional evaluation before and during pegvaliase

#### 3.2.1 Baseline

The baseline nutritional status of patients before pegvaliase initiation is necessary to evaluate treatment outcomes. In general, clinical evaluation and follow-up should be in agreement with the recommendations of the European PKU guidelines [2]. The consensus group proposed a standard of nutritional status assessments before the initiation of pegvaliase (Table 2). Regular assessment of blood Phe concentrations and anthropometric parameters is required to determine the efficacy of pegvaliase and evaluate the impact of dietary changes on the patient's anthropometrics, respectively. It is also recommended to extensively assess nutritional intake and food patterns (e.g., total protein intake, protein substitute intake, natural protein intake, quality of the protein source, number of meals each day) to determine the adequacy of protein and nutrient intake. Pre-pegvaliase, patients may typically fall into one of three categories: 1) the patient is not following a restricted diet and is meeting the dietary reference intake (DRI) for natural protein of 0.8 g/kg, 2) the patient is following a Phe-restricted diet with an adequate amount of protein equivalent from protein substitutes to meet protein needs or 3) the patient is consuming an inadequate amount of protein equivalent from protein substitute and not eating sufficient natural protein. A food frequency questionnaire, as has been described by Viau et al. 2014 [15], combined with either 24-h dietary recall or 3-day food records is the usual method of assessing nutritional intake. Innovative methods for the assessment of dietary intake can also be explored, such as photographic meal diaries or filling out dietary information via a mobile application. Food neophobia questionnaires are useful to assist dietitians and/or psychologists/counsellors in assessing the patients' willingness/readiness and fears in changing their eating habits. While not validated in patients with PKU specifically, a modified version of the Food Neophobia Scale from Pliner et al. [16] has been previously used in a prospective controlled study to assess neophobia in patients with PKU [17,18]. As pegvaliase may change the patient's dietary regimen and result in decreased nutrient intake from non-natural sources, the biochemical micronutrient and amino acid status, including tyrosine, should be assessed. Although some EU dietitians commonly assess body composition of their adult PKU patients, this is not usually evaluated in US dietary practices. Therefore, analysis of body composition would be an optional requirement to initiate pegvaliase. Nevertheless, collecting data on body composition would aid metabolic teams in understanding the impact of dietary changes associated with pegvaliase treatment on PKU-associated comorbidities, such as obesity, metabolic syndrome, and the risk of cardiovascular disease and hyperlipidaemia [[19], [20], [21]]. Due to pegvaliase being a treatment for late adolescent and adult patients with PKU monitoring of blood pressure, insulin resistance and lipid profile is recommended [22]. In addition, lifestyle and quality of life (QoL) questionnaires, such as the PKU-QoL [23,24], may be valuable to examine healthy or unhealthy changes in lifestyle and the overall impact of pegvaliase on the physical, emotional and social aspects of PKU patients.

**Table 2:** Proposed assessments for nutritional status. Frequency of all assessments may be increased if there is a clinical reason for concern or according to the centre's current follow-up protocol.

Assessment	Details	Frequency during titration and dosing optimisation	Frequency during maintenance and diet normalisation
***Consensus group recommendations for****minimal****nutritional status assessments***			


Blood Phe concentrations	- Primary biomarker of blood Phe control	- Monthly regular follow-up	- Monthly regular follow-up
		- Every 2 weeks in case of LBP (blood Phe <30 μmol/L)	- Every 2 weeks in case of LBP (blood Phe <30 μmol/L)
		- More frequently upon the metabolic team's discretion	- More frequently upon the metabolic team's discretion
Anthropometric evaluation	- Weight, height and BMI	- Up to once per month	- Every 6 months
	- Waist circumference		
Nutritional intake assessment	- Food frequency questionnaire (preferred)	- Once per month	- Every 6 months
	- 24-h recall records or 3-day food records		
	- Food neophobia questionnaire (optional)		

***Assessments for nutritional status evaluation****strongly recommended****by the consensus group***			


Blood analysis (in addition to Phe)	- Full amino acid profile, including tyrosine	- Every 6 months	- Annually
		- Tyrosine concentrations assessed with blood Phe	- Tyrosine concentrations assessed with blood Phe
	- Prealbumin		
	- Full blood cell count		
	- Functional markers of micronutrients		
	• Ferritin		
	• Total homocysteine or methylmalonic acid		
	- Micronutrients		
	• Folic acid		
	• Vitamin B12		
	• Vitamin D		
	• Iron		
	• Calcium		
	• Zinc		
	• Selenium		


***Assessments for nutritional status evaluation****recommended****by the consensus group***			


Body composition analysis	- Fat-free mass and body fat	- Every 6 months or once yearly	- Once yearly
	- Phase angle obtained from bioelectrical impedance analysis (optional)		

Bone mineral density	- DXA scan to exclude osteopenia and osteoporosis	- Every 3–5 years	- Every 3–5 years
	- Per EU PKU guidelines, this should be done based on identified risk, but not essential in routine follow-up [2]		

BMI: body mass index; DXA: dual-energy x-ray absorptiometry; LBP: low blood Phe, Phe: phenylalanine, PKU: phenylketonuria.

#### 3.2.2 Titration and dose optimisation phase

As indicated by the US and EU pegvaliase prescription labels, blood Phe should be assessed monthly during titration and dosing optimisation phases [12,13]. However, the consensus group suggests more frequent blood Phe monitoring upon the metabolic team's discretion. Centres with a more frequent follow-up protocol in place before adoption of pegvaliase, may either reconsider or continue their blood Phe monitoring frequency when offering pegvaliase, reflecting the patients' individual needs and support required. According to the EU pegvaliase prescription label, it is recommended to monitor blood Phe levels every two weeks in the event of low blood Phe (LBP; i.e., <30 μmol/L) [13]. The clinical trials have shown that prolonged periods of LBP may occur but the implications are unclear, thus requiring close monitoring of blood Phe levels to understand if LBP is related to any side effects [14]. Generally, the consensus group would advocate more frequent blood Phe monitoring than indicated in the prescription labels when this is clinically indicated. During the titration and dosing optimisation phase, nutritional intake should ideally be assessed with each blood Phe measurement, using a tool of the dietitian's choice (Table 2).

#### 3.2.3 Maintenance phase

Once the dietary treatment is relaxed (as described in section 3.3.2.) during the pegvaliase maintenance phase, patient evaluation every 6 months in clinic to assess adherence and treatment outcomes is recommended. At minimum, blood Phe should be monitored monthly, as per EU prescription label [13]. Blood Phe may be assessed more frequently upon the metabolic team's discretion, e.g., during periods when protein intake and/or pegvaliase dose are adjusted. The frequency of nutritional intake assessments may be decreased to once every 6 months together with the assessment of anthropometric parameters. If resources are available, micronutrient, amino acid and body composition analyses can be performed annually or on a more regular basis if necessary, particularly when managing comorbidities [20,21]. In general, all nutritional status assessments should be performed more often if there are any clinical concerns or if the treatment centre already follows a stricter protocol, assuming this will not cause an excessive burden for the patient.

### 3.3 Treatment goals of pegvaliase

#### 3.3.1 Blood Phe target range

Longo et al., described that it is feasible for patients on pegvaliase to maintain blood Phe concentrations below the usual lower target range of 120 μmol/L and within the physiological range of 31–120 μmol/L for healthy adults, when taking a normal protein intake without protein substitute [14]. It was stressed that it is still important to avoid persistent LBP or hypophenylalaninemia (<30 μmol/L). This recommendation was constructed from expert opinion and data that was collected during the pegvaliase clinical trial programme spanning over a decade, demonstrating that approximately 50% of patients were able to achieve blood Phe levels ≤120 μmol/L after 24 months of treatment [11,14]. The experts in our group who had personal experience of using pegvaliase with adults with PKU supported and agreed with the recommendation of Longo et al. [14]. However, some experts in our group needed further research with 24-h blood Phe profiling data before recommending a lower blood Phe target of 31–120 μmol/L. They suggest, data are needed to assess the diurnal fluctuations and variations in blood Phe, ensuring that blood Phe concentrations can be maintained within the physiological range in patients who are able to normalise their diet with pegvaliase. As a consequence, we have made no recommendation on the lower blood Phe target. Nevertheless, future guidance will be provided when additional studies and real-world experience address the amino acid profile, bone mineral density and growth outcomes of pegvaliase-treated patients achieving long-term blood Phe concentrations between 31 and 120 μmol/L while being on a normal diet. For the upper blood Phe target, the majority of the experts recommended targeting concentrations below 240–360 μmol/L. Although there is no published evidence in adults, recent data suggest that blood Phe concentrations below 240 μmol/L may improve neurocognition in children/adolescents with PKU [25,26].

#### 3.3.2 Normalisation of diet

##### 3.3.2.1 Recommended protein intake

The consensus group defined a normal protein intake as at least 0.8 g of protein/kg/day from a mix of animal and plant sources, without any supplementation from protein substitutes, while ensuring that all nutrient requirements are met. This protein intake provides the recommended intake of protein defined by the World Health Organization [27] and should be sustained over time with the maintenance of adequate clinical and biochemical nutritional status. The dietitian is essential to ensure that these recommendations are met. Additionally, the dietitian should advise patients to eat high-quality biological protein, which may be challenging for patients who have not been used to eating these foods prior to treatment with pegvaliase. For patients with PKU on a vegan diet, the protein intake should be, in general, 20% more (1 g of protein/kg/day), due to the intake of potentially lower quality protein. Currently, there is insufficient knowledge to make a recommendation on the upper limit for protein intake, especially in patients with high body mass index (BMI). The dietitian's primary focus should be on increasing diet quality and concomitantly prevent any increase in body weight that results in the onset or worsening of overweight or obesity. Although no robust evidence exists demonstrating the real causes of overweight in PKU, increasing diet quality of patients with PKU should be viewed as an opportunity to optimise body composition as well as gut microbiota, with increasing evidence indicating this is unbalanced in dietary-treated patients with PKU [28,29]. A healthy diet with regular physical exercise should always be emphasised, as previously suggested [19].

##### 3.3.2.2 Adjustment of diet and protein intake during treatment with pegvaliase

**Fig. 1:**
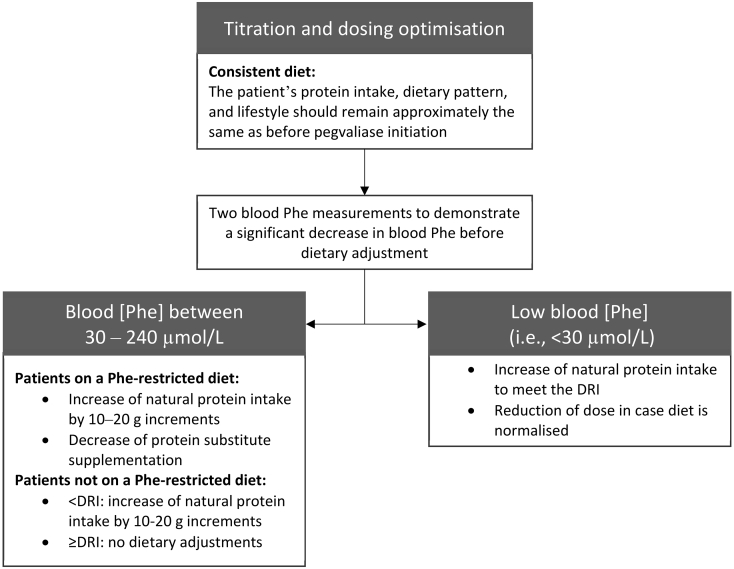
Consensus group recommendations for adjustment of diet and protein intake during treatment with pegvaliase. The dietary reference intake (DRI) is defined as 0.8 g of protein/kg/day from a mix of animal and plant sources, excluding protein substitute supplementation.

The dietitian should counsel that patients maintain a consistent diet and protein intake during the introduction and titration of pegvaliase to determine treatment response [14]. In the pegvaliase clinical trial protocols, patients maintained their natural and medical protein intake within ±10% from baseline [11]. As this is not a realistic goal in clinical practice, the consensus group defined a consistent diet as follows: “The patient's protein intake, dietary pattern and lifestyle should remain approximately the same as before pegvaliase initiation” (Fig. 1). During the pegvaliase dose optimisation phase, it is recommended to increase natural protein intake when blood Phe concentrations are between 30 and 240 μmol/L in patients on a Phe-restricted diet. Natural protein intake should be increased following two low blood Phe measurements (Fig. 1). Natural protein should be increased in increments of 10–20 g, considering the comfort level of the patient (Fig. 1). Patients on a Phe-restricted diet may be hesitant about relaxing natural protein intake by large increments and may encounter several challenges when increasing natural protein intake, e.g., taste, texture and smell of foods, cost, cooking, choosing non-nutritious foods or poor-quality protein. Therefore, it is necessary to review the patients' expectations, offering them education and counselling to ensure any individualised plan of introducing protein is implemented. Also, when increasing the intake of natural protein, the protein equivalent from protein substitute should be assessed and reduced if necessary (Fig. 1). For patients on a regular, protein-containing diet prior to starting pegvaliase, protein adjustments will be dependent on the baseline nutritional status of the individual patient and usual protein intake. If the patient does not meet the recommended protein intake (i.e., 0.8 g of protein/kg/day from a mix of animal and plant sources, excluding protein substitute supplementation), natural protein intake can be increased. Patients should be encouraged to eat high-quality protein to ensure balanced nutrition. When the nutritional needs are met and blood Phe remains within the treatment target range, no adjustments to the dietary protein intake should be made in the first phase of pegvaliase treatment (Fig. 1). In case natural protein intake is above the DRI for protein, dose reduction can be considered if the patient is well-controlled on pegvaliase (Fig. 1). In the event of LBP, patients should be advised to increase natural protein intake. When diet is normalised, dose reduction should be considered. Regardless of the dietary or dose adjustments, blood Phe concentrations should be monitored closely to evaluate the impact of changes in natural protein intake or dose on the outcomes of pegvaliase.

#### 3.3.3 Additional treatment goals

Additional treatment goals of pegvaliase are improvements in neurocognition and (neuro)psychological outcomes, QoL and emotional well-being, together with the minimisation of comorbidities, such as obesity and metabolic syndrome [19]. These additional goals emphasise the importance of a multidisciplinary team (Table 1), including a psychologist/counsellor, in the management of patients with PKU on pegvaliase.

### 3.4 Education

Education of patients before treatment with pegvaliase and during the transition to a normalised diet is crucial to ensure correct use of pegvaliase and appropriate dietary management. One-on-one counselling should continue to be the mainstay but can be complemented with additional educational tools to inform and support patients with PKU before and during treatment with pegvaliase. Printed educational materials, such as information leaflets/handouts, remain important but may not be sufficient to convey the message, as patients are not always reading or understanding these. Other tools enabling patient education may include instructional videos, workshops and mobile applications for (self-)monitoring diet and pegvaliase injections [30].

Dietitians should be educated on the nutritional challenges of patients treated with pegvaliase. Although the interaction between dietitian and patient may be reduced after diet normalisation from once monthly to semi-annually, it remains important to maintain this central point of contact for follow-up of comorbidities and long-term diet ensuring patients continue to eat an adequate diet meeting the nutritional needs of each patient and for re-education of pegvaliase-treated patients. Moreover, dietary guidance is essential in patients who may discontinue pegvaliase, e.g., adverse events. This includes counselling the patient on how to return to diet when pegvaliase is not an option. Furthermore, Longo et al. recommend discontinuation of pegvaliase use at least 4 weeks prior to a planned pregnancy [14]. Therefore, nutrition counselling is essential to avoid hyperphenylalaninemia throughout (pre)pregnancy, after which rechallenge of pegvaliase can be successfully implemented [31].

## 4 Conclusions

This practical road map was developed to provide initial guidance for dietitians, who have an essential role in the management of patients on pegvaliase. The recommendations were developed by dietitians who had previously treated adult PKU patients with pegvaliase together with insights from dietitians and one paediatrician who have experience in treating PKU patients in Europe. Because only one EU dietitian had previous experience with using pegvaliase, guidance on the clinical approach was mainly adapted from US experience. Combining the outcomes of a face-to-face data-review meeting and a virtual consolidation meeting, consensus recommendations were formed among the PKU experts on the composition of the core pegvaliase treatment team, the nutritional evaluation and management of patients on pegvaliase, treatment goals and on education of dietitians and patients using pegvaliase. A summary of the dietitian road map recommendations can be found in Table 3.

**Table 3:** Summary of the dietitian road map recommendations developed by the consensus group.

Topic	Recommendation(s) by the consensus group
Multidisciplinary team	The core treatment team should comprise a physician, dietitian/nutritionist and a nurse. If resources are available, also a psychologist/counsellor (highly recommended), social worker, home support worker and coordinator should be part of the multidisciplinary team
Nutritional status evaluation	- Blood Phe concentrations, anthropometrics and nutritional intake were identified as minimally required for follow-up
	- Blood Phe concentrations should be assessed monthly (or every 2 weeks in case of low blood Phe, <30 μmol/L), but may be monitored more frequently upon the metabolic team's discretion, e.g., when changing protein intake or pegvaliase dose
	- Nutritional intake is ideally monitored with each blood Phe measurement, preferably using a food frequency questionnaire in combination with 24-h recall or 3-day food records
	- Practical recommendations for additional nutritional assessments can be found in Table 2

Treatment goals of pegvaliase	- Pegvaliase should aim to maintain blood Phe levels below 240 μmol/L but more evidence on the safety of achieving physiological blood Phe levels will need to be collected before making any recommendation on the lower limit of the blood Phe target range
	- A normalised diet: at least 0.8 g of protein/kg/day (DRI for protein intake) from a mix of animal and plant sources, without any supplementation from protein substitutes, while ensuring that all nutrient requirements are met
	- Additional goals: improvements in neurocognition and (neuro)psychological outcomes, quality of life and emotional well-being

Adjustment of diet and protein intake	- When starting pegvaliase, the patient should be on a consistent diet, defined as: “The patient's protein intake, dietary pattern and lifestyle should remain approximately the same as before pegvaliase initiation”
	- An increase in natural protein intake can be considered when blood Phe concentrations are below 240 μmol/L but should be individualised
	- Natural protein should be increased by increments of 10–20 g per day
	- If the patient is taking protein substitutes, these should be reduced proportionally if this is possible, once natural protein intake is increased
	- Protein intake of vegan patients should be generally 20% more, but nutritional status needs to be calculated on an individual basis
	- In obese patients, care should be focussed on diet and exercise to reduce weight

Education	Patient education before and during treatment with pegvaliase is crucial, with a focus on nutritional intake and correct use of pegvaliase

DRI: dietary reference intake; Phe: phenylalanine.

As these recommendations are based on expert opinion and available literature only, they are intended to provide initial recommendations to dietitians. In the future, these dietary recommendations will be updated based on additional evidence and increasing experience from the metabolic teams.

## Funding

The face-to-face and virtual meeting leading up to this dietitian road map, as well as assistance in development of the current manuscript, were funded by BioMarin International, Ltd.

## Declaration of Competing Interest

All authors received financial reimbursement from BioMarin at fair market value for their participation in the face-to-face and/or virtual meeting. The manuscript was developed by the authors and reviewed by BioMarin.

Outside the submitted work, the authors disclose the following. Bausell H received personal fees from BioMarin, Ultragenyx, Horizon and Vitaflo. Bélanger-Quintana A reports personal fees from BioMarin, Nutricia, Vitaflo, Orphan Europe, Takeda and Genzyme. Rocha JC received research grants from BioMarin, Glutamine and Cambrooke, as well as personal fees from BioMarin, Applied Pharma Research, Nutricia, Merck Serono, Vitaflo, Cambrooke, PIAM and Lifediet. MacDonald A reports research funding from BioMarin, Nutricia, Applied Pharma Research, Vitaflo, Galen, Metax, Mevalia and Arla, as well as lecture fees from BioMarin, Applied Pharma Research, Nutricia and Vitaflo, and consultancy fees from BioMarin, Applied Pharma Research, Arla, Nutricia and Vitaflo. Met Ed reports grant funding from BioMarin, Nutricia, Vitaflo and Horizon Pharmaceuticals. Bernstein L and Rohr F report lecture fees from Vitaflo.
